# Controlling Spin Interference in Single Radical Molecules

**DOI:** 10.1021/acs.nanolett.2c05068

**Published:** 2023-04-18

**Authors:** Yahia Chelli, Serena Sandhu, Abdalghani H. S. Daaoub, Sara Sangtarash, Hatef Sadeghi

**Affiliations:** Device Modelling Group, School of Engineering, University of Warwick, Coventry CV4 7AL, United Kingdom

**Keywords:** molecular electronics, single stable radical, quantum transport, spin interference, electrical conductance

## Abstract

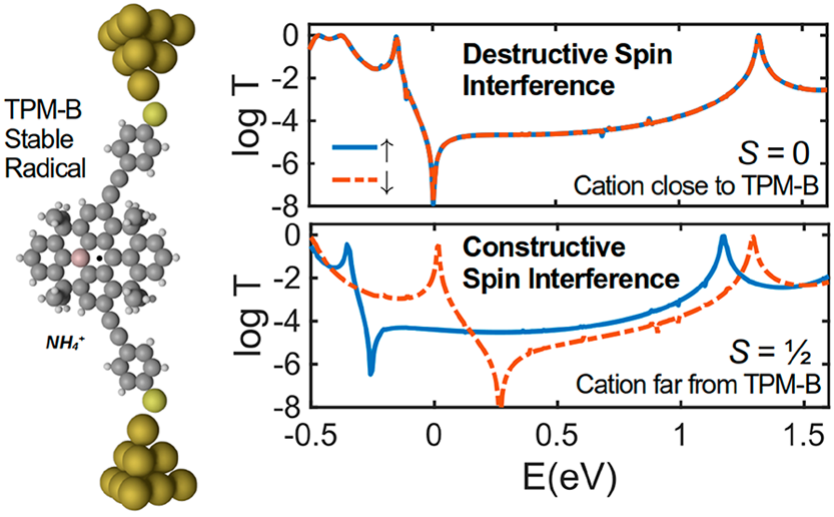

Quantum interference (QI) dominates the electronic properties of single molecules even at room temperature and can lead to a large change in their electrical conductance. To take advantage of this for nanoelectronic applications, a mechanism to electronically control QI in single molecules needs to be developed. In this paper, we demonstrate that controlling the quantum interference of each spin in a stable open-shell organic radical with a large π-system is possible by changing the spin state of the radical. We show that the counterintuitive constructive spin interference in a *meta*-connected radical changes to destructive interference by changing the spin state of the radical from a doublet to a singlet. This results in a significant change in the room temperature electrical conductance by several orders of magnitude, opening up new possibilities for spin interference based molecular switches for energy storage and conversion applications.

Most molecules have an even number of electrons, with each orbital holding a pair of electrons with opposite spins.^1^ However, free radicals have an unpaired electron, which makes the molecule unstable, highly reactive, and sensitive to components in the atmosphere. This makes the processing and measuring of their physical properties and theoretical modeling challenging. However, in stable radical molecules, the unpaired electron is structurally protected, which makes them stable from a few moments to several years. An example of a very stable organic radical is tris(2,4,6-trichlorophenyl)methyl (TTM).^2^

Stable radicals have recently attracted interest for their exotic electronic and spintronic properties and their exceptional performance in electronic devices such as batteries, transistors, and light-emitting diodes.^3−9^ For example, molecules with radical side groups show spin filtering,^10^ and the nitroxide radical side group is predicted to enhance the electrical conductance and Seebeck coefficient and suppress the thermal conductance of single-molecule junctions, which makes it a promising candidate for thermoelectric energy generation or cooling.^11^ The simultaneous enhancement of the room-temperature electrical conductance and Seebeck coefficient has also been reported for Blatter radical single molecule junctions.^12^ Redox-addressable Verdazyl radicals show bias-dependent rectification and promise a single molecule transistor-like switching even at room temperature.^13^ Moreover, Kondo resonance has been observed in polychlorotriphenylmethyl radicals.^1,14^

Due to their small size and atomically precise structure, molecules exhibit quantum effects such as quantum interference (QI) even at room temperature.^15−20^ This has been demonstrated recently in closed-shell polycyclic aromatic hydrocarbons (PAHs).^20,21^ For example, *meta*-connected pyrene shows a low conductance, which is a sign of destructive QI (DQI). The DQI in *meta*-connected anthraquinone (AQ) changes to constructive QI when the charge state of AQ changes, leading to an increase in conductance.^22^ Other examples include QI in single molecules with benzene, naphthalene, and anthracene cores.^23,24^ DQI is expected in these closed-shell PAHs when there is an odd number of carbon atoms between the two connection points to electrodes.

Unlike closed-shell PAHs, QI can be different for the majority and minority spins in stable PAH radicals, resulting in different interference patterns for each spin (spin interference, SI). In this paper, we demonstrate a counterintuitive spin interference effect in a boron-stabilized planar neutral π-radical and show that both destructive (DSI) and constructive (CSI) spin interference are possible in PAH radicals. Furthermore, we demonstrate that for a stable radical with a fixed chemical structure, a transition from spin 1/2 to spin 0 (*S* = 1/2 ↔ *S* = 0) changes the counterintuitive SI to the conventional QI. As a result, the electrical conductance is tuned by more than 2 orders of magnitude in this stable radical. To explain the counterintuitive SI in stable radicals, we developed a generic Hückel model which can be used to exploit the electronic properties of radicals.

Figure 1a shows the molecular structure of the boron-stabilized planar neutral π-radical, triphenylmethyl (TPM-B). TPM-B is shown to be thermally stable and resistant to atmospheric conditions due to its considerable spin delocalization.^25^ In addition to its stability at room temperature, the graphene-like molecular structure of TPM-B makes it an ideal molecule to study spin interference effects because it has multiple possible connection points to electrodes. For these reasons, we studied the electronic structure and spin transport properties of TPM-B when it is connected to metallic electrodes through thiol anchors and acetylene linkers from three different connection points (Figure 1b and c). Our spin density calculations show that the spin density is mainly delocalized on the TPM-B core, with the largest density on the central ring consisting of the boron and radical carbon atoms (Figure 1d). This is in agreement with previous studies.^25^ Thiol anchors were chosen because they have high binding energy to electrodes (*E*_B_ = 2.1 eV) and make good contacts to gold electrodes experimentally.^26^ To demonstrate that spin interference can be different in these radicals compared to their closed-shell counterparts, we also studied a closed-shell analogue of TPM-B where the boron atom was replaced with a carbon atom (TPM in Figure 1b).

**Figure 1:**
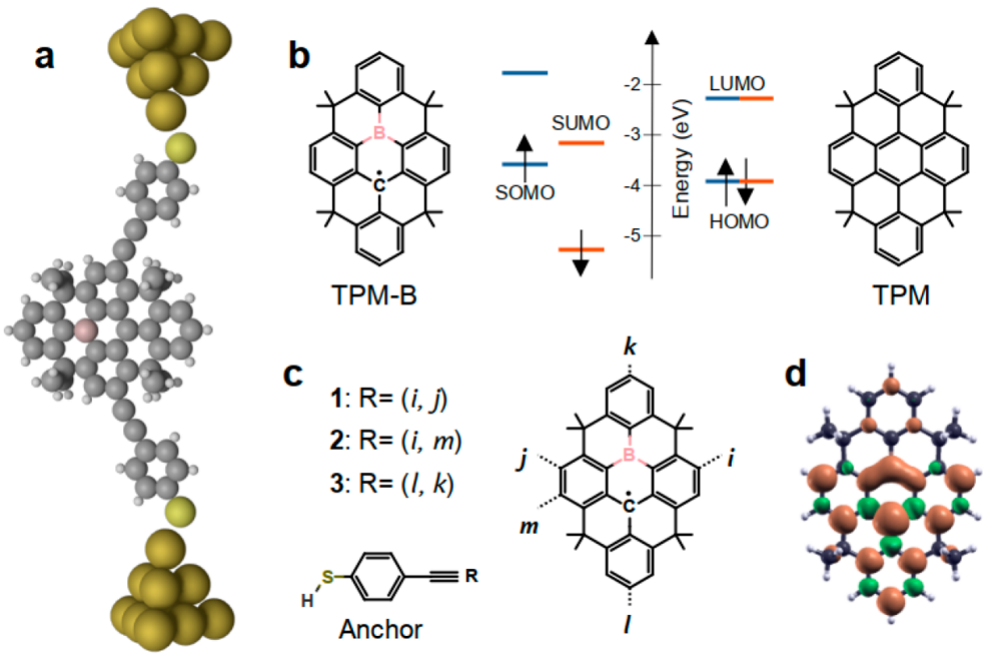
Molecular structure of boron-stabilized triphenylmethyl (TPM-B) radical. (a) Schematic of a single molecule junction formed by the TPM-B molecular core, thiol anchors, and acetylene linkers between the anchor and molecular core. (b) The energy level diagram for TPM-B radical and its counterpart closed shell molecule TPM. (c) TPM-B core with three connection points (*i, j*), (*i, m*), and (*l, k*) to anchors and the molecular structure of a thiol anchor with an acetylene linker. Molecules **1**–**3** are formed by connecting the molecular core to the anchor from R point. (d) Spin density distribution in the TPM-B core.

To study the quantum spin transport through junctions formed by TPM-B molecular cores **1**–**3** and their closed-shell analogues (TPM **1′**–**3′**), we first found the ground-state geometries of each junction using the SIESTA^27^ implementation of Density Functional Theory (DFT). Figure 1a shows TPM-B with molecular core **1** in a junction formed by two gold electrodes. The junction configurations with molecular cores **2**, **3**, and TPM **1′**–**3′** are shown in Figure S1. We then obtained the spin-polarized mean-field Hamiltonian of each junction from DFT and combined it with our transport code GOLLUM^28,29^ to calculate the transmission coefficient *T*^σ^(*E*) for the majority (spin-up, *σ=↑*) and minority (spin-down, *σ=↓*) spins traversing from one gold electrode to the other.

Figure 2 shows *T*^σ^(*E*) of molecules **1**–**3** and their closed-shell analogues (**1′**–**3′**) between gold electrodes for the spin-up (blue curve) and spin-down (red curve) electrons. The spin-up transport through molecule **1** is dominated by DSI, which is characterized by a sharp antiresonance around *E* = −0.1 eV (between the first blue resonances at either side of *E* = 0 eV in Figure 2a). However, the spin interference for spin-down electrons is constructive (red curve in Figure 2a). In contrast, a destructive QI is expected for both spin-up and spin-down electrons for the closed-shell analogue of molecule **1** (molecule **1′** obtained by replacing B with a C atom), as shown in Figure 2b.

**Figure 2:**
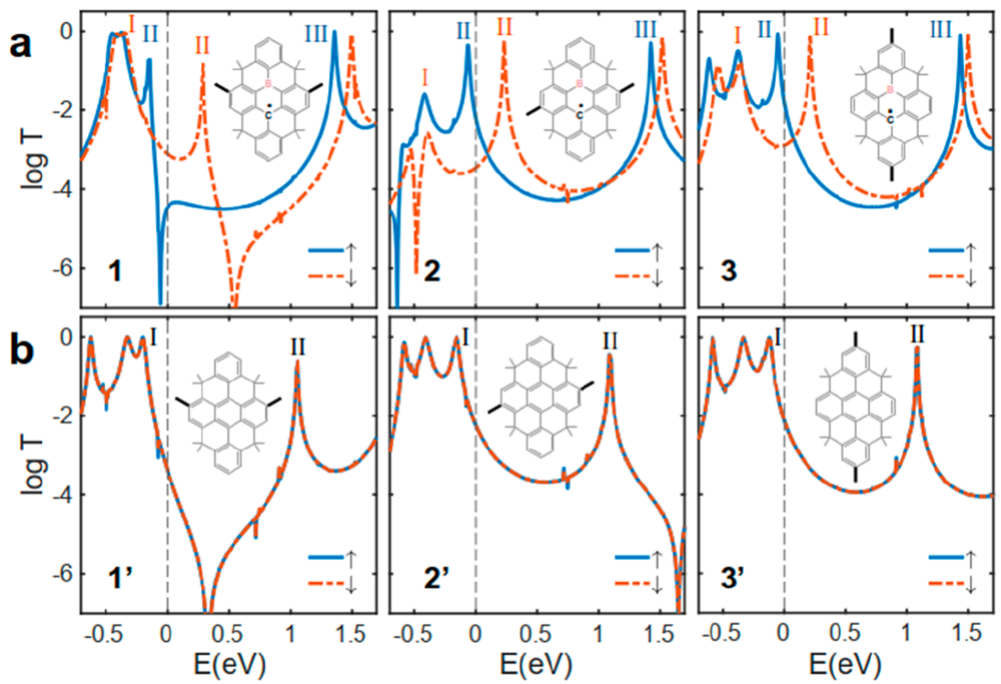
Quantum transport through TPM and TPM-B. (a) Spin-dependent DFT-transmission coefficient of TPM-B with molecular core **1**–**3** and (b) their closed-shell TPM counterpart **1′**–**3′**. Inset shows the molecular cores. The thick black line shows the connection points to electrodes. Each of these molecular cores is connected to the gold electrodes through anchors (Figure 1a). *E* = 0 eV denotes the DFT Fermi energy. Note that the transport resonances labeled I, II, and III correspond to the orbitals shown in Figure 3.

To understand the counterintuitive spin interference pattern in molecule **1**, we calculated the spin orbitals of the TPM-B core for the minority and majority spins (Figure 3a). Our calculations show that the highest occupied molecular orbital (HOMO) for spin-up (also called SOMO) is almost identical to the lowest unoccupied molecular orbital (LUMO) for spin-down (also called SUMO) as shown in Figure 3a. Similarly, HOMO–1 for spin-up is similar to HOMO for spin-down. This means that the molecular orbitals for spin-down are shifted up in energy by one level compared to spin-up (see more levels in Figures S2–S5). As a result, frontier orbitals for spin-up and spin-down are different. From the orbital rules^29,30^ (eq 1), we would then expect a different interference pattern for an electron with spin-up compared to that of spin-down. This is because the transmission coefficient between sites *i* and *j* (*T*_*ij*_(*E*)) is proportional to the Green’s function *g*_*ij*_ of a molecule, defined as
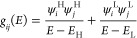
1In this expression, ψ_*b*_^*a*^ is the wave function at site *b* for state *a*, *E*_*a*_ is the energy level associated with this state, and *a*= H, L denotes HOMO and LUMO, respectively. From eq 1, it is clear that *g*_*ij*_ will only vanish at a certain energy (e.g., *E* = (*E*_L_*+E*_H_)/2, the middle of the H–L gap) if the signs of the products ψ_*i*_^H^ψ_*j*_^H^ and ψ_*i*_^L^ψ_*j*_^L^ are the same. For example, at the middle of the H–L gap:
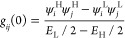
2

**Figure 3:**
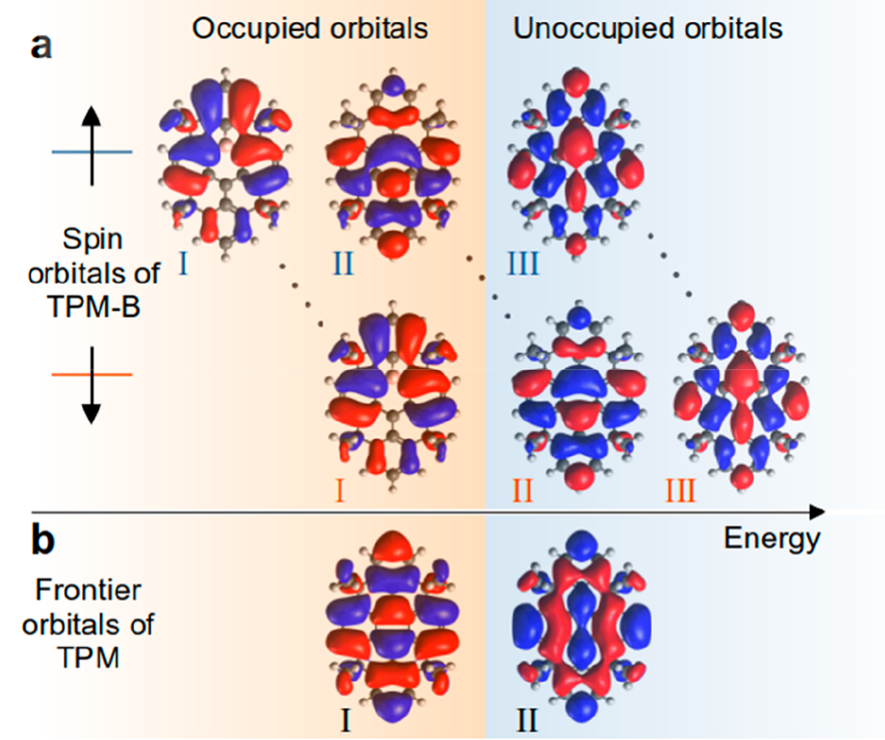
Frontier molecular orbitals of TPM and TPM-B. (a) Frontier molecular orbitals of TPM-B core for spin up and spin down electrons, and (b) frontier molecular orbitals of TPM.

If a molecule is connected to the electrodes from connection points *i* and *j* (see Figure 1c), the product of the wave function signs at sites *i* and *j* for the spin-up HOMO is positive (note that blue and red colors represent the sign of the wave function and the size of it represents the amplitude of the wave function in Figure 3), while it is negative for the spin-up LUMO. This means that *g*_*ij*_ (and consequently *T*_*ij*_) can vanish, leading to DSI. However, the product of the wave function signs at sites *i* and *j* is different for the spin-down HOMO and LUMO. Therefore, CSI is expected for spin-down for this connection point. This is reflected in the DFT transmission functions for **1** (Figure 2a), where only the spin-up transmission shows an antiresonance.

For *para*-connected **2** and **3**, the SI between HOMO and LUMO is expected to be constructive for both spins from the orbital rules (Figure 3a). This is indeed the case, as shown by the transmission coefficients of **2** and **3** in Figure 2a. This is in contrast to the QI observed in molecules with radical side groups.^10,31^ We attribute this to the more extended spin density in radicals with a large π-system compared to that in radical side groups. Figure 2b shows the transmission coefficients of **1′–3′**, the closed-shell analogues of **1**–**3**. A conventional QI pattern is observed in **1′–3′**, with the *meta*-connected **1′** showing DQI and the *para*-connected **2′** and **3′** showing CQI. This is also consistent with the orbital rule analysis using the molecular orbitals of TPM (Figure 3b). The molecular orbitals of **1′**–**3′**, including the anchor groups, are shown in the Supporting Information and agree with the analysis based on the molecular core alone.

A simple one-orbital-per-atom Hückel model cannot explain these counterintuitive spin interference results. We need to use a two-orbital-per-atom Hückel model and develop a way to parametrize it. In what follows, we present a simple generic tight-binding (Hückel) model for radicals that can be used to predict spin interference. We start by building simple tight-binding (TB) Hamiltonians for spin-up and spin-down. Our aim is to parametrize the TB spin-up and spin-down Hamiltonians so that the resulting molecular orbitals for each spin match those predicted by DFT calculations. We find that the DFT orbitals (Figure 3a) are reproduced when we set all on-site energies and coupling integrals the same for both spin-up and spin-down Hamiltonians, except for the on-site energy of the atom with the main radical character, which we set to ε_↑,↓_ = ε_1_ ± ε_2_ where ε_1_ is the same for both spin-up and spin-down Hamiltonians and ε_2_ is positive for one spin and negative for the other.

Using this simple rule, we set ε_radical carbon_= ε_1_ ± ε_2_ (gray site in Figure 4a, e.g., ε_1_ = 0.7 eV and ε_2_ = 0.7 eV). All other parameters are set using the conventional Hückel model, with all on-site energies of carbon atoms with sp^2^ hybridization set to ε_0_ = 0 eV, carbon atoms with sp^3^ hybridization set to large values (e.g., ε_4_=-5.5 eV), and the boron heteroatom set to ε_3_ = 0.5 eV, for both spin Hamiltonians. The TB model built based on this approach is in good agreement with DFT results, as shown in Figure 4. For example, the TB orbitals (Figure 4b) are in good agreement with the DFT orbitals for each spin (Figure 3a), and the resulting TB transmission coefficients shown in Figure 4c are also in good agreement with the corresponding DFT transmissions in Figure 2a. Therefore, to build a Hückel model for radicals, we need to (1) build two Hamiltonians, one for each spin, and (2) set all parameters in the spin Hamiltonians to be the same, except for the on-site energy of the atom with the main radical character which should be set to ε_rad_^↑^ = ε_1_ + ε_2_ and ε_rad_^↓^ = ε_1_ – ε_2_. Using this TB model, the DFT orbitals, interference pattern, and corresponding transmissions are reproduced in radicals.

**Figure 4:**
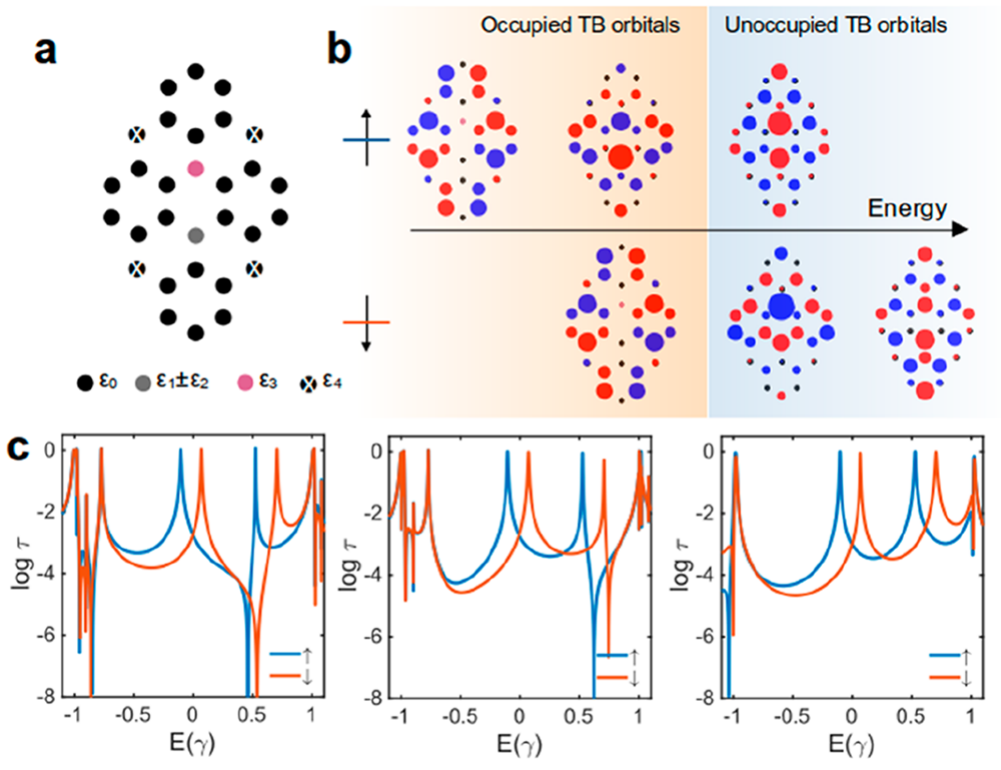
Tight-binding (TB) model of TPM-B radical. (a) Structure of TB model with one orbital per spin per atom. Black, gray, purple, and hashed black circles denote sp^2^ carbon, radical carbon, boron, and sp^3^ carbon atoms with on-site energies ε_0_, ε_1_ ± ε_2_, ε_3_, and ε_4,_ respectively. All parameters are the same for spin-up and spin-down Hamiltonians except the on-site energy of radical carbon (gray atom) which is ε_1_ + ε_2_ for spin-up Hamiltonian and ε_1_–ε_2_ for spin-down Hamiltonian. (b) TB molecular orbitals for spin-up and spin-down electrons, (c) Spin-dependent transmission coefficient for TB TPM-B molecular core connected to TB electrode from similar connection points to **1**, **2**, and **3**, respectively. The transport resonances on either side of *E* = 0 in c correspond to the highest occupied spin orbital and the lowest unoccupied spin orbital in b.

So far, we have discussed counterintuitive interference in radical molecules. Next, we aim to demonstrate that the spin interference in TPM-B is controlled by its spin state. This leads to more than 2 orders of magnitude change in electrical conductance. To demonstrate this, we calculated the spin transmission coefficient through **1** (Figure 1a) as a function of distance (*d*) between an ammonium cation and the TPM-B core (Figure 5a) using DFT and scattering theory (see Methods). When the ammonium cation is far from the TPM-B core (*d* > 10 Å), **1** remains in the spin state *S* = 1/2 and retains its radical character. As shown in Figure 5b, the spin transmission coefficients and spin interference pattern for the majority and minority spins are very similar to those of **1** in the absence of the ammonium cation (Figure 2a). By decreasing *d*, the spin state of TPM-B changes from *S* = 1/2 at *d* > 10 Å to *S* = 0 at *d* = 2.6 Å. This change in the spin state leads to changes in the spin transmission coefficient (see Figure 5b) and spin interference for spin-down.

**Figure 5:**
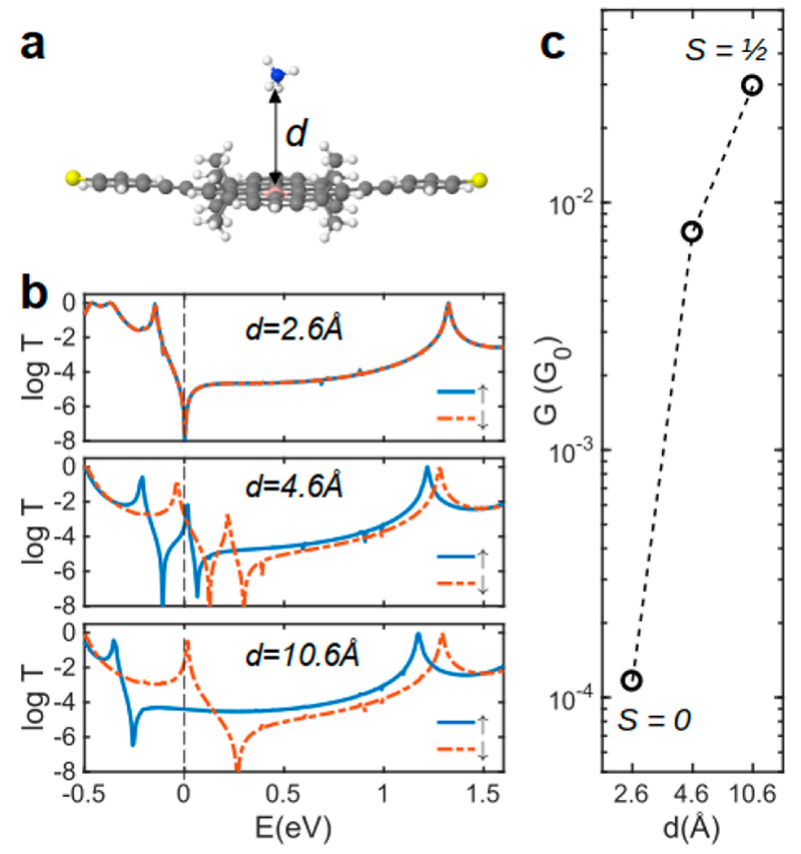
Controlling spin interference and spin transition (*S* = 1/2 ↔ *S* = 0) using ammonium cation in TPM-B radical. (a) Molecular structure of TPM-B radical interacting with an ammonium cation. (b) DFT transmission coefficient for molecule 1 (Figure 1a) between electrodes as a function of distance (*d*) between the ammonium cation and the TPM-B backbone. (c) Room temperature electrical conductance as a function of *d* at DFT Fermi energy.

As shown in Figure 5b, the spin-up and spin-down transmissions become identical when the ammonium cation is close to the TPM-B core (*d* = 2.6 Å), resulting in a clear antiresonance in *T*(*E*) at the DFT Fermi energy and low electrical conductance at room temperature (Figure 5c). Figure S8 shows the effect of spin state tuning on the SI pattern and *T*(*E*) with smaller *d* intervals. The orbital rule also supports the changes in the spin interference pattern, as shown by our molecular orbital calculations in the presence of an ammonium cation in Figure S9. We have also repeated this calculation in the presence of an ammonium cation and a hydroxide anion and obtained similar results (see Figure S10). To demonstrate that the changes in conductance are robust against the junction configurations to electrodes and changes in the position of ions on the TPM-B backbone, we have also performed calculations with various junction conformations (see Figure S11). The calculated conductance histograms show that the most probable conductance for junctions with *d* = 10.6 Å is about 2 orders of magnitude higher than that of junctions with *d* = 2.6 Å.

The SI pattern changes with the spin state of TPM-B because the degenerate spin HOMO level in the *S* = 0 spin state (Figure S9) splits into two new states (SOMO (spin-up) and SUMO (spin-down)) in the *S* = 1/2 spin state (Figure S3). This means that the spin-down orbital loses an electron and moves above the Fermi energy. As a result, the spin interference is dominated by different frontier orbitals for each spin, which changes the spin interference pattern. Since the conductance is the sum of the transmissions for spin-up and spin-down electrons (see Methods), the total transmission increases significantly when the spin interference pattern for spin-down electrons changes from DQI to CQI as a result of changes in the spin state of TPM-B from *S* = 0 to *S* = 1/2.

This provides a route for in situ control of spin interference and electrical conductance in organic stable radicals. This is a generic feature of radicals and can be used to design spin switches that operate at room temperature. This is an advantage compared to QI in closed-shell PAHs because controlling QI in situ has been proven to be difficult in closed-shell molecular structures.^15^ Despite several attempts, changes in the QI pattern have only been possible through the synthesis of a series of molecules with different connection points to electrodes or heteroatom substitutions.^20,32,33^ Different interference patterns for each spin in radicals, and the possibility to change them by modifying their spin state, provide a valuable tool for in situ control of electrical conductance and promise new avenues for molecular switches that operate at room temperature.

In summary, we demonstrated that spin interference in radical molecules with a large π-system is different from that in closed-shell molecules. Our results show that spin interference in radicals is controlled by their spin state and that the spin interference can be switched from destructive to constructive by changing the spin state of a boron-stabilized planar neutral π-radical from a doublet to a singlet. This results in a significant change in electrical conductance. This strategy could be used to electronically switch on and off the electrical conductance and opens up new possibilities for spin interference-based molecular switches that operate at room temperature.

## Computational Methods

The Hamiltonian of the structures described in this paper was obtained using density functional theory (DFT) or constructed from a simple tight-binding model as described below.

### TB Model

The spin-dependent Hamiltonian is constructed as  where σ = ↑,↓ denotes spin-up and spin-down, respectively, and the coupling integrals are γ_*i*,*j*_^σ^ = −1, the on-site energies are the same for both spin Hamiltonians except the on-site energy of the radical carbon . The parameters used for spin Hamiltonian are ε_1_ = ε_2_ = 0.7, , and ε_B_^σ^ = 0.5. These parameters reproduce DFT orbitals for each spin and transmission coefficient as discussed in the main text.

### DFT Calculation

The geometry of each structure studied in this paper was relaxed to the force tolerance of 10 meV/Å using the *SIESTA*(27) implementation of DFT, with a double-ζ polarized basis set (DZP) and the Generalized Gradient Approximation (GGA) functional with Perdew–Burke–Ernzerhof (PBE) parametrization. A real-space grid was defined with an equivalent energy cutoff of 250 Ry. We then calculate spin polarized molecular orbitals and spin density of gas phase molecules.

### Spin Transport

To calculate the electronic properties of the device, from the converged DFT calculation, the underlying spin polarized mean-field Hamiltonian *H*^σ^ was obtained where σ = *↑, ↓* and *↑* (*↓*) denotes majority (minority) spin. *H*^σ^ was combined with our quantum transport code, *GOLLUM*.^28^ This yields the spin-dependent transmission coefficient *T*^σ^(*E*) for electrons of energy *E* (passing from the source to the drain) via the relation *T*^σ^(*E*) =  where  describes the level broadening due to the coupling between left L and right R electrodes and the central scattering region, Σ_L,R_^σ^(*E*) are the retarded self-energies associated with this coupling and  is the retarded Green’s function, where *H*^σ^ is the Hamiltonian and *S* is the overlap matrix obtained from *SIESTA* implementation of DFT. The total transmission is then calculated from *T*(*E*) = (*T*^*↑*^ + *T*^*↓*^)/2.

### Electrical Conductance

Using the approach explained in ref (29), the electrical conductance is calculated from Landaure’s formula , where *f* is the Fermi–Dirac probability distribution function, *T* is the temperature, *E*_F_ is the Fermi energy, *G*_0_ = 2*e*^2^/*h* is the conductance quantum, *e* is the electron charge, and *h* is the Planck’s constant.

### Conductance Histograms

To construct conductance histograms shown in Figure S11, we follow the same procedure as in ref (26). First, we form a series of junctions with different contacting modalities to electrodes and calculate the electrical conductance *G* for a range of electrodes Fermi energies *E*_F_. Next, we create the conductance histograms using the calculated conductance for each junction and for a wide range of *E*_F_ between the frontier transport resonances. The peaks in the conductance histograms are fitted with a log-normal distribution and their center is defined as the most probable conductance.

## Data Availability

The input files to reproduce simulation data can be accessed by contacting the authors.

## Supporting Information Available

Details of the calculations, molecular orbitals for all molecules, spin densities, additional transmission coefficient calculations, and conductance histograms (PDF)
