# Supplementary material for: Controlling Spin Interference in Single Radical Molecules
Source: Nano Lett. 2023 Apr 18;23(9):3748–53. doi: 10.1021/acs.nanolett.2c05068 (PMC10176569; doi:10.1021/acs.nanolett.2c05068)
Supplement: Supplementary file 1 — nl2c05068_si_001.pdf [file nl2c05068_si_001.pdf]

Supporting Materials

Controlling Spin Interference in Single Radical Molecules

Yahia Chelli, Serena Sandhu, Abdalghani H. S. Daaoub, Sara Sangtarash and Hatef Sadeghi*

Device Modelling Group, School of Engineering, University of Warwick, CV4 7AL Coventry, United Kingdom

* *hatef.sadeghi@warwick.ac.uk*

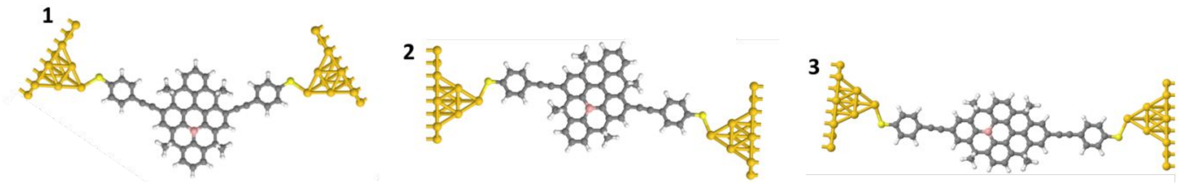

Figure S1: Junction formed by molecules **1**, **2**, and **3** with TPM-B core between gold leads.

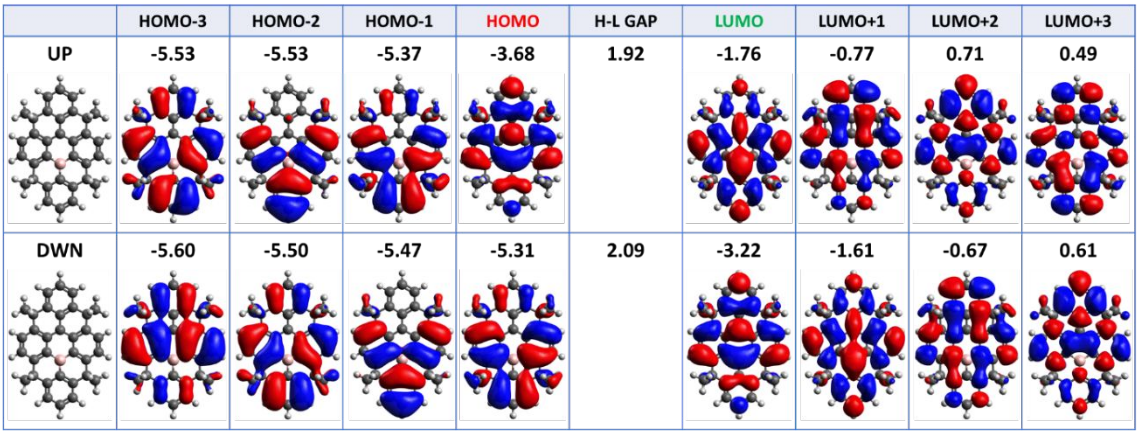

Figure S2: Spin-up and spin-down DFT orbitals with corresponding energy values for the TPM-B core molecule.

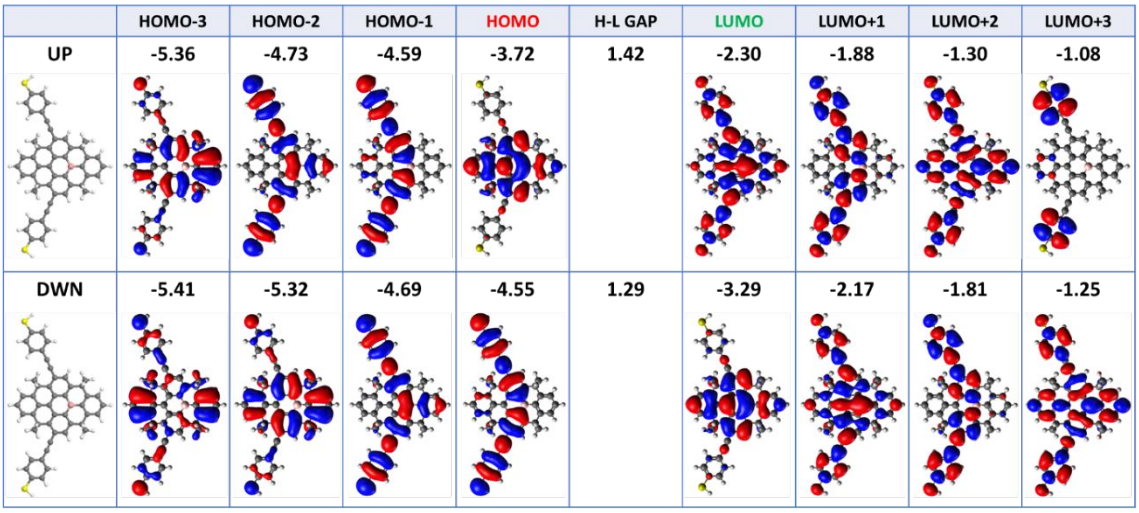

Figure S3: Spin-up and spin-down DFT orbitals with corresponding energy values for **1**.

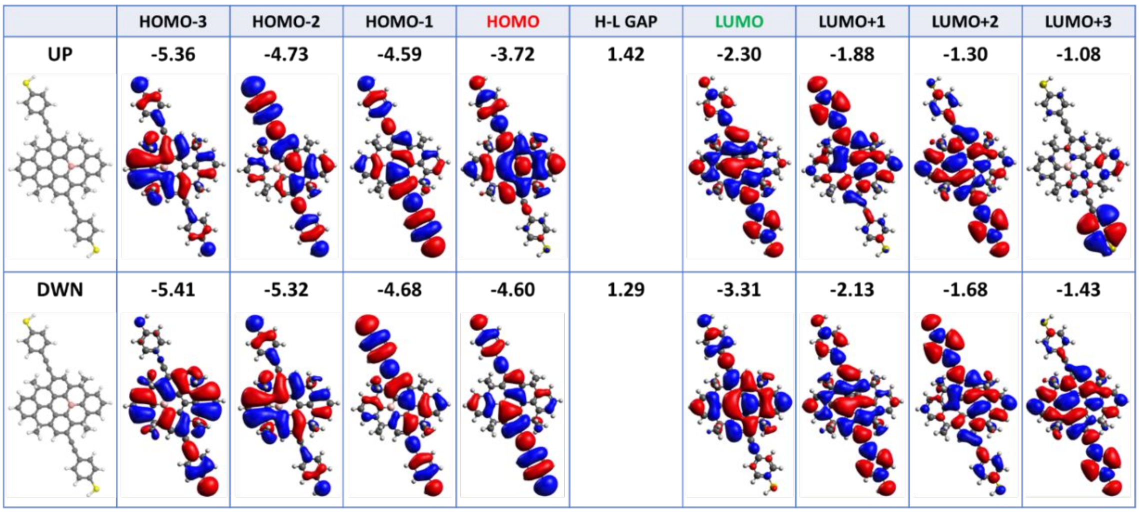

	HOMO-3	HOMO-2	HOMO-1	HOMO	H-L GAP	LUMO	LUMO+1	LUMO+2	LUMO+3
UP	-5.36	-4.73	-4.59	-3.72	1.42	-2.30	-1.88	-1.30	-1.08
DWN	-5.41	-5.32	-4.68	-4.60	1.29	-3.31	-2.13	-1.68	-1.43

Figure S4: Spin-up and spin-down DFT orbitals with corresponding energy values for **2**.

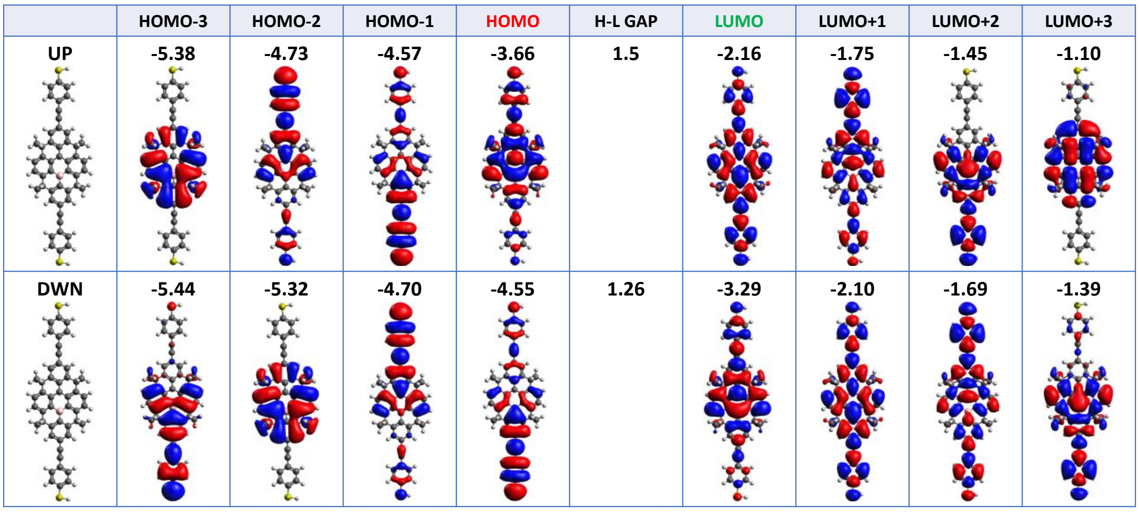

	HOMO-3	HOMO-2	HOMO-1	HOMO	H-L GAP	LUMO	LUMO+1	LUMO+2	LUMO+3
UP	-5.38	-4.73	-4.57	-3.66	1.5	-2.16	-1.75	-1.45	-1.10
DWN	-5.44	-5.32	-4.70	-4.55	1.26	-3.29	-2.10	-1.69	-1.39

Figure S5: Spin-up and spin-down DFT orbitals with corresponding energy values for **3**.

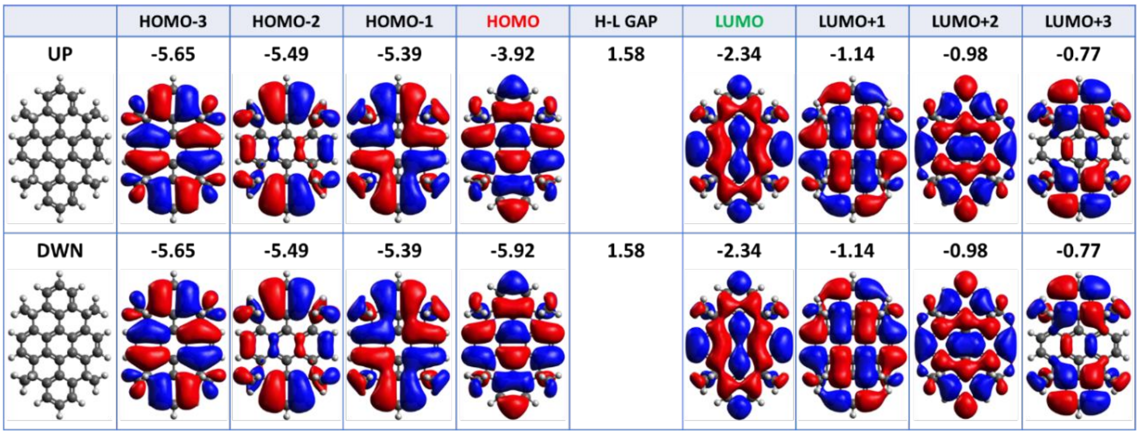

	HOMO-3	HOMO-2	HOMO-1	HOMO	H-L GAP	LUMO	LUMO+1	LUMO+2	LUMO+3
UP	-5.65	-5.49	-5.39	-3.92	1.58	-2.34	-1.14	-0.98	-0.77
DWN	-5.65	-5.49	-5.39	-5.92	1.58	-2.34	-1.14	-0.98	-0.77

Figure S6: Spin-up and spin-down DFT orbitals with corresponding energy values for TPM core molecule.

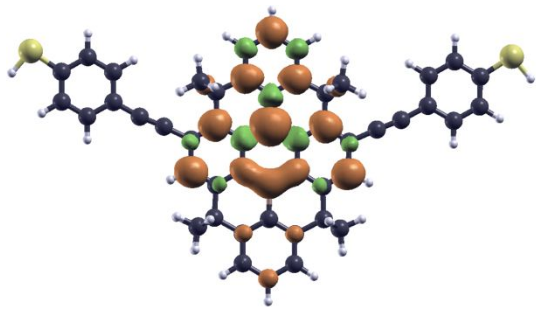

Figure S7: Spin density of **1**.

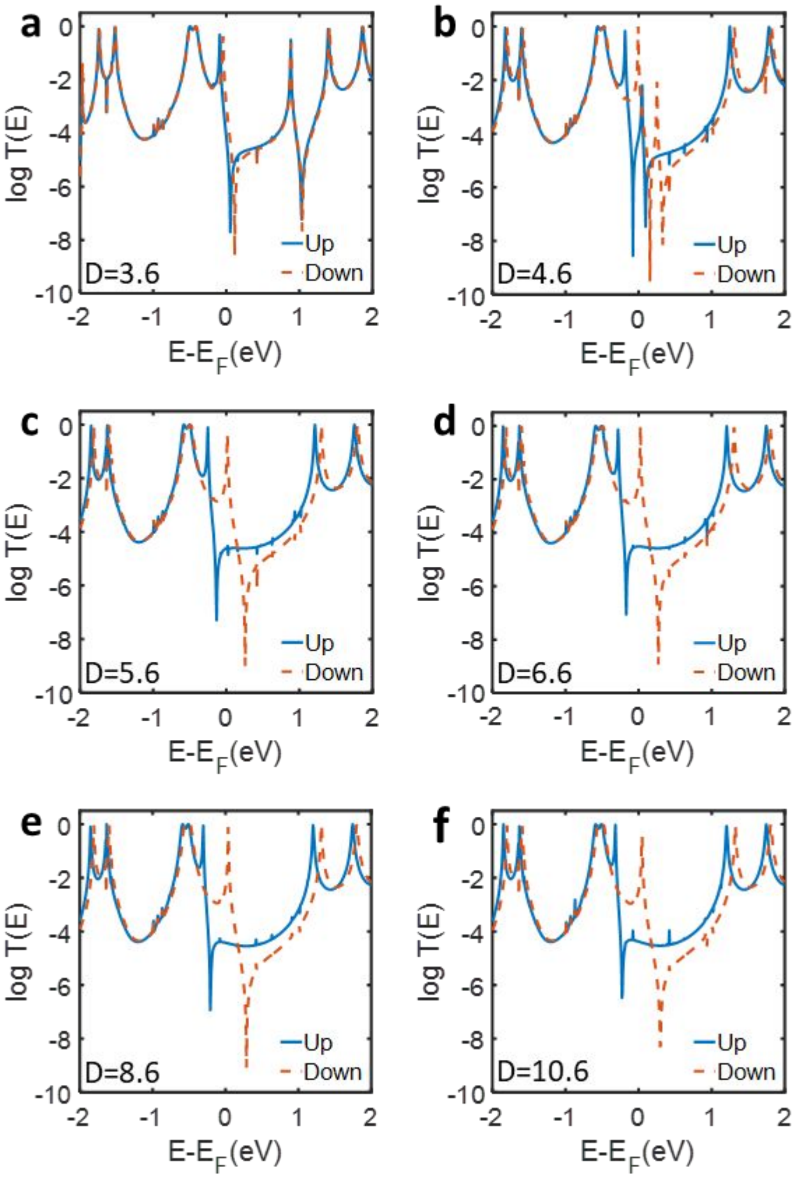

Figure S8: Spin transmission functions of **1** in the presence of ammonium cation located at distance D from TPM-B backbone. D is equal to 3.6, 4.6, 5.6, 6.6, 8.6, and 10.6 in **a-f**, respectively.

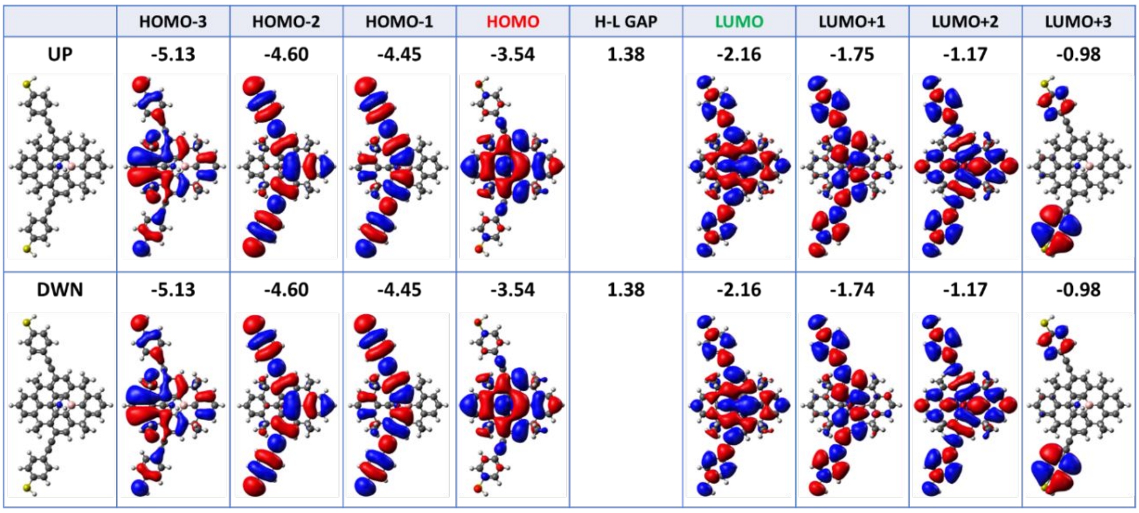

Figure S9: Spin-up and spin-down DFT orbitals with corresponding energy values for **1** in the presence of NH4 cation at optimum position from the TPM-B core (2.6Å).

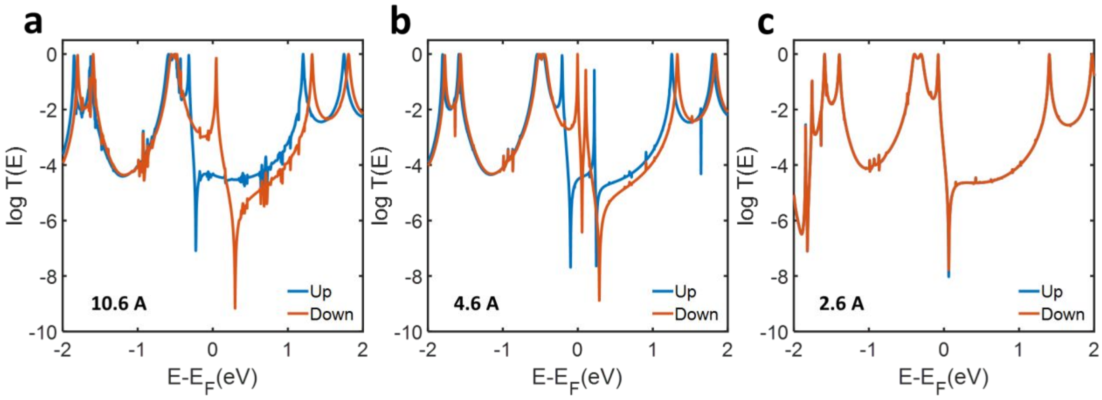

Figure S10: Spin transmission functions of **1** in the presence of a pair of OH^- and NH_4^+ located at distance *d* from the TPM-B backbone. *d* is equal to 10.6, 4.6, and 2.6 in **a**-**c**, respectively.

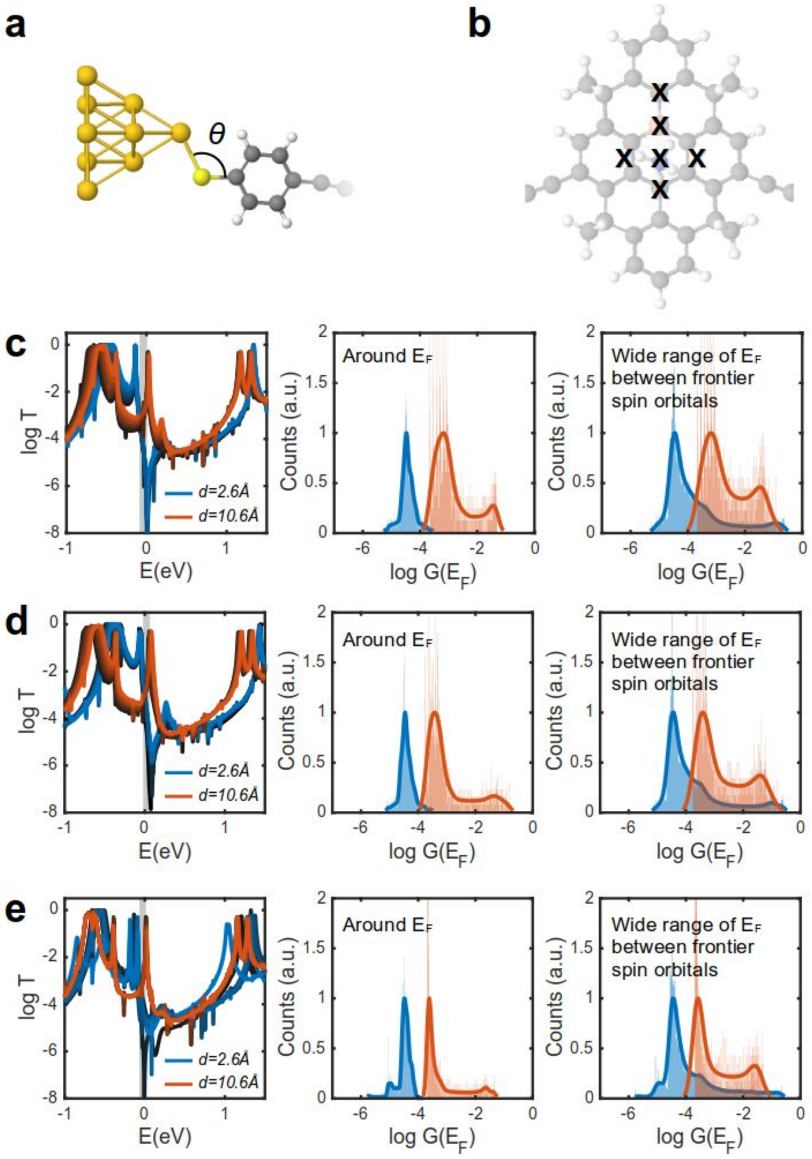

Figure S11. Conductance histograms of 1 as a function of junction conformation. a. Changes in electrode – molecule conformation by varying the angle between them, **b.** varying the position of an ion on the TPM-B backbone. Transmission coefficients and calculated conductance histograms for TPM-B **c.** with an ammonium cation and **d.** with a pair of an ammonium cation and a hydroxide anion placed in two different distances (*d*=2.6Å and *d*=10.6Å) from the TPM-B backbone and as a function of changes of *θ*. To construct this, we change *θ* from its ground state configurations by 30° with 5° intervals and produce a series of junctions and calculate the transmission coefficient for each configuration, **e.** transmission coefficients, and calculated conductance histograms for TPM-B with an ammonium cation placed in two different distances (*d*=2.6Å and *d*=10.6Å) and as a function of cation position on the TPM-B backbone as shown in **b**.
